# Flow cytometric immunophenotyping (FCI) of lymphoma: correlation with histopathology and immunohistochemistry

**DOI:** 10.1186/1746-1596-3-43

**Published:** 2008-11-06

**Authors:** Abeer M El-Sayed, Mohammad H El-Borai, Abeer A Bahnassy, Shadia MS El-Gerzawi

**Affiliations:** 1 Pathology Department, National Cancer Institute, Cairo University, Cairo, Egypt

## Abstract

**Background:**

To evaluate the role of flow cytometric immunophenotyping (FCI) in diagnosis and characterization of lymphoma tissue specimens from Egyptian patients.

**Methods:**

FCI using 2 and 3 color staining approaches, was performed on 50 fresh lymph nodes specimen from Cairo NCI patients with suspected lymphoma presenting with either localized or generalized lymphadenopathy.

FCI results were correlated with histopathologic as well as immunophenotypic[by immunohistochemistry (IHC)] findings.

**Results:**

By FCI, cases were diagnosed as follows: 9(18%) reactive hyperplasia (RH), 32(64%) B-cell non-Hodgkin's lymphoma (B-NHL) [24 diffuse large (DLBCL), 2 follicular, 3 small lymphocytic, 2 mantle cell lymphoma and a case of T cell rich B cell lymphoma], 3 (6%) T cell NHL [2 peripheral T cell lymphoma and a case of anaplastic large cell lymphoma], 2(4%) Hodgkin's lymphoma (HL) while 4 (8%) were non-lymphomatous tumors (NLT). Light chain restriction (LCR) was detected in the 32 FCI diagnosed B-NHL. The overall concordance between FCI versus histopathology and IHC was 88%. The sensitivity and specificity of FCI in diagnosis of NHL was 94.9% and 100% respectively; in HL they were 40% and 100% respectively and in NLT, both sensitivity and specificity were 100% while for RH were 100% and 89.1% respectively.

**Conclusion:**

FCI is a sensitive and specific method in diagnosis and classification of NHL as well as in detection of monoclonality. False negative results could be due to the presence of heterogeneous populations of lymphocytes in special types of lymphoma.

## Introduction

Lymphoma represents one of the major health problems allover the world. It is a common malignancy affecting both children and adults and is continuing to increase rapidly. In the Middle East, non-Hodgkin's lymphoma (NHL) has a high incidence contributing to 7% of total cancer [1] as compared to 4% in USA [2].

In Egypt, lymphoma represented 11.66% of all diagnosed cancer cases at the NCI-Cairo University during the period 2003–2004 according to the Cancer Pathology Registry, with non-Hodgkin's lymphoma constituting 76.5% of these cases. The B-phenotype comprised 81.1% while T-phenotype represented 9.8% while 9.1% of the cases were non-specified [3].

The advent of immunophenotyping of samples from patients with lymphoproliferative disorders has added much to proper diagnosis and classification and better understanding of the pathogenetic mechanisms underlying the development of these disorders [4]. In the context of lymphoma diagnosis, immunohistochemistry (IHC) has the advantage that the cells of interest are identified morphologically and it is applicable retrospectively to fixed tissues, though fixation might lead to loss of some cells and/or cellular antigenicity [5]. However, only a single marker is routinely used on tissue sections, which permits examination of about 100 cells only. Moreover, there is a reported difficulty in demonstrating immunoglobulin light chains [6].

On the contrary, flow cytometry (FCM) allows a more precise definition of individual cell types since the cells of interest are identified by a combination of physical characteristics and the use of multiple antibodies directly conjugated with different fluorochromes. It also has the ability to assess monoclonality through detection of immunoglobulin light chain expression and the results can be available within few hours after receiving the specimen [7-9].

In addition, flow cytometric immunophenotyping (FCI) has become a widely used laboratory procedure for diagnosis and sub-typing of lymphoma. It is an objective and quantitative diagnostic tool that allows quick multiparametric analysis of a very large number of cells (20.000–50.000 cells per sample) which could be obtained from small tissue sample (0.1 cm^3 ^or even smaller). Meanwhile, analysis of such small samples is facilitated by applying dual & triple markers that permit in a single experiment, the detection of expression of combination of 2 or 3 antigens respectively on the same cell [10,11]

Several studies have supported the usefulness of FCI in diagnosing lymphoma in fine needle aspiration (FNA) samples as well as in staging and follow up of cases [12-14].

However, only few reports are available regarding the role of FCM in tissue diagnosis and typing of lymphoma. Morse et al. [15] reported that 9 out of 16 cases (56%) were diagnosed by FCI alone as lymphoma or carcinoma and 4 (25%) were consistent with a final diagnosis of normal or reactive hyperplasia whereas, 3 cases only had histologic evidence of malignancy on biopsies that escaped detection by FCM. Moreover, Dunphy, [16] reported that FCI data contributed significantly to, or was consistent with the final tissue diagnosis in 94% of a large series including 373 cases. Furthermore, Martinez et al. [17] reported that FCI diagnosed 218 cases of NHL out of 250 cases with negative predictive value 0.52 and positive predictive value 1.

In 2000 mayall et al.[18], Dunphy [19] reported that FCI in combination with touch imprint cytomorphology was useful in excluding diagnosis of lymphoma and non lymphomatous tumors & showed 100% concordance with histopathologic results.

Regarding Ig light chain detection by FCM, Leers et al. [20] estimated the clonality of lymphoproliferative disorders by FCI & IHC. They pointed out a major drawback of immunohistochemical detection of monoclonality in B-cell lymphoproliferative disorders which was the lack of contrast between surface-immunoglobulin staining and extracellular immunoglobulin staining. Monoclonality was established in 9 out of 10 NHL cases by FCI while only 6 of 9 cases were conclusive by IHC.

The present study was conducted to assess the diagnostic role of FCM for immunophenotyping of lymphoma on freshly excised tissue biopsies by comparing the results of FCI to routine histopathology and immunohistochemistry.

## Methods

The study included 57 freshly excised lymph node specimens obtained from patients who attended to the National Cancer Institute clinics, Cairo University during the period 2003–2006 complaining of localized or generalized lymphadenopathy. The mean age of patients was 47.7 (range 5–72 years).

Specimens were immediately suspended in sterile chilled RPMI-1640 tissue culture medium. Each specimen was divided into three parts, the first part was used for the preparation of touch imprints stained by modified PAP to assess the presence of malignant lymphoma cells and facilitate diagnosis, the second was used to prepare single cell suspension by mechanical dispersion for FCI, and the third was put in 10% neutral buffered formalin, embedded in paraffin and processed routinely for histologic diagnosis and immunohistochemistry for lymphoma sub-typing.

### Flow cytometric immunophenotyping (FCI)

### Immunohistochemistry (IHC)

 FCI results were correlated with histopathologic / immunohistochemical  immunophenotyping results  performed on paraffin-embedded material according to standard protocols using the following antibodies (all from Dako Ltd., Cambridge, UK): CD45 (LCA, T29), CD20 (L-26), CD79acy (HM57), CD3 (PC3), CD45RO (UCHL1), CD5 (CD5/45/F6), CD4 (OPD4), CD8 (C8), CD10 (SS2/36), CD56 (NK1), CD15, CD30, ALK-1.

#### Preparation of a single cell suspension

Cells from affected nodes were dispersed by squeezing the tissues against nylon or metal mesh (pore size 50–70 μm) into a glass beaker. Tissues were always kept wet by adding sterile phosphate buffered saline solutions (PBS) or serum to maintain cell viability. The suspension was then centrifuged 4–5 min at 1200–1400 rpm, the supernatant was discarded and the cell pellet was resuspended in 2 ml PBS. Part of the suspension was used for assessing cell viability by staining with trypan blue and cell counting [14]. The percentage viability was calculated, the required total number is around 10^6^/ml and the optimum acceptable percent of viable cells is about 80% [21].

The rest of the suspension was subjected to staining for FCI analysis. Cell surface antigen staining was performed using specific directly conjugated fluorochrome-labeled monoclonal antibodies which were assembled in panels according to case requirement. The monoclonal antibodies used were CD45, CD45RO, CD 20, CD19, CD3, CD10, CD5, CD23, CD30, CD15, bcl-2, anti-κ, anti-λ, CD4 and CD8 [6].

#### Antibody panels design

Isotype control IgG1-FITC/IgG2a-PE was used at the start of each run. This was followed by staining with the screening triple marker; CD3-FITC/CD19-PE/CD45-PerCP. Further analysis with the appropriate antibodies was performed on the remaining of the sample based on the initial results of both touch imprints and the screening triple marker. The markers used in this study (All from BD Bioscience, USA) were: **1) **The dual markers (CD5-FITC/CD19-PE; Anti kappa-FITC/CD19-PE; Anti lambda-FITC/CD19-PE; CD4-FITC/CD8-PE), and **2) **The single markers [CD20-FITC; CD10-FITC; CD45RO-PE; CD23-PE; CD30-FITC; CD15-FITC; Anti-BCL2-FITC].

FCM quality control including alignment, calibration, and color compensation was performed before sample acquisition according to manufacture's instructional manual.

#### Staining for FCI analysis

Staining was performed according to the manufacturer's instructions (Becton Dickinson, Bioscience, USA). Briefly, 20 μl of each of the used monoclonal antibodies were added to 100 μl of the cell suspension in separate tubes and mixed by vortexing. This was followed by 30 min incubation at room temperature (RT) in the dark, washed twice in 2 ml of PBS and centrifugation for 5 min at 300 × g. Then 0.5 ml of 1% paraformaldehyde was added for fixation and samples were acquired on the FCM after 30 min [22].

#### Acquisition

Acquisition and analysis of stained suspension was performed by FACScan flow cytometer (Becton Dickinson, USA) acquiring at least 20.000 – 30.000 cells at a high rate of 400–500 cells/second for each marker. Negative isotype control was run first to identify the position of the negative and the positive populations. At least two plots were drawn during the acquisition of each tube; one of them displayed forward scatter (FSc) on X axis versus side scatter (SSc) on Y axis to identify the size and granularity of cells and to exclude debris and dead cells. The second plot displayed the antibody marker on X axis versus FSc or the other marker in case of dual markers on Y axis.

#### Data storage

Data was stored in the list-mode where the raw data for each parameter on every analyzed cell were sequentially stored in a list to allow reanalysis at any time including redrawing gates, different population gating and new histogram drawing.

#### Sample analysis

Analysis of sample tubes was performed as follows: setting cursors for differentiating positive and negative populations (Isotype control plot analysis) so that ≥ 98% of the cells are negative. The tube containing CD45 (gating reagent) was analyzed first to set a gate around lymphocyte clusters using FSc and SSc patterns and fluorescence staining based on low FSc and SSc patterns and bright stain of lymphocytes with CD45.

Light-scattering patterns were examined on each sample tube and the remaining sample tubes were analyzed with the cursors previously set based on the isotype control. The data was reported as a percentage of the total lymphocytes and/or percentage of gated population. Absolute numbers of positive and negative populations were also reported.

#### Interpretation of data

The Size of cells was defined depending on FSc as: small (FSc 200–400), medium (FSc 400–600) or large (FSc > 600) [21]. The fluorescence intensity was determined by using a log scale of dot plot as follows: the population which lies in region 10°-10^1 ^was considered negative, region 10^1^-10^2 ^was considered weak, region 10^2^-10^3 ^was considered moderate and region > 10^3 ^was considered strong.

#### Detection of B-cell monoclonality

This was determined by overlay of histograms of both anti-kappa and anti-lambda and measuring the difference between them using the Cell Quest software of the machine. Light chain restriction (LCR) was considered when κ/λ was < 0.5 or κ/λ was > 2.5 or if there is absence of both κ & λ expression by tumor cells [19]. A negative (isotype) reagent control was used with each specimen to determine non-specific binding of the mouse monoclonal antibody to the cells and to allow setting of markers for distinguishing fluorescence-negative and fluorescence-positive cell populations. The marker set on the negative control plot was copied on each analyzed plot of dual markers plots, so dividing it into 4 quadrants.

Analysis of the population in each quadrant was as follows: the lower left quadrant; double negative to both markers, the lower right quadrant; positive for X axis marker only, the upper left quadrant; positive for Y axis marker only, and the upper right quadrant; positive to both markers.

### Statistical methods

Sensitivity and specificity of FCI as compared to histopathologic findings were calculated. Sensitivity is defined as the probability of a positive test among patients with disease. Specificity is the probability of a negative test among patients without disease. The positive predictive value (PPV) and the negative predictive value (NPV) were also measured [23].

$$Sensitivity=\frac{\text{Number of true positive cases}\times \text{1}00}{\text{Number of true positive cases}+\text{False negative cases}}$$

$$Specificity=\frac{\text{Number of true nagative cases}\times \text{1}00}{\text{Number of true negative cases}+\text{False positive cases}}$$

## Results

Out of the 57 biopsies received, 50 were evaluated by both FCM and IHC and 7 were not suitable for evaluation by FCM due to reduced viability of cells below 85%. Thus only these 50 cases were used in all statistical evaluation & correlation of results.

### The results of touch imprint

The stained touch imprints were evaluated as regards their contribution to the FCI data and their role in facilitating the diagnosis of cases. The cases were divided into 4 groups: **1) **7 cases (14%) showed small mature lymphocytes, suggestive of reactive hyperplasia (RH); **2) **37 cases (74%) showed variable features suggestive of NHL of which, 26 (52%) showed large atypical lymphoid cells with vesicular nuclei and prominent nucleoli associated with atypical small to medium sized cells, 8 (16%) revealed atypical small lymphoid cells and 3 (6%) revealed atypical lymphocytes of varying size with irregular nuclear membranes, necrotic debris and multinucleated cells; **3) **Two cases (4%) revealed few large atypical mononuclear cells and large bi-nucleated cells with prominent nucleoli in a background of small lymphocytes suggestive of Hodgkin's lymphoma (HL) and **4) **4 cases (8%) showed non-lymphomatous malignant cells with scattered or few lymphocytes, suggestive of non lymphomatous or metastatic tumor (MT) (Table 1).

**Table 1:** Correlation between touch imprints, flow cytometric immunophenotyping, routine histology and immunohistochemical findings.

Group	Touch imprints No. (%)	FCI No. (%)	Histopathology & Immunohistochemistry No. (%)
RH	7 (14)	9 (18)	4 (8)

NHL	37 (74)	35 (70)	37 (74)
B-cell		32 (64)	34 (68)
T-cell		3 (6)	3 (6)

HL	2 (4)	2 (4)	5 (10)

MT	4 (8)	4 (8)	4 (8)

Total	50 (100)	50 (100)	50 (100)

### Flow cytometric (FCI) findings

Positive reaction of cells to monoclonal antibodies was detected as fluorescent dots in the dot plot quadrants. The distribution of dots differed depending on the number of markers used and the status of positivity to each marker. The diagnostic distribution of the 50 specimens according to FCI was as follows: 9 cases (18%) were diagnosed as RH, 32 cases (64%) were B-cell NHL, 3 cases (6%) were T-cell NHL, 2 cases (4%) were HL and 4 cases (8%) were diagnosed as non-lymphomatous (NLT) or metastatic tumors (MT) (Table 1).

**Figure 1:**
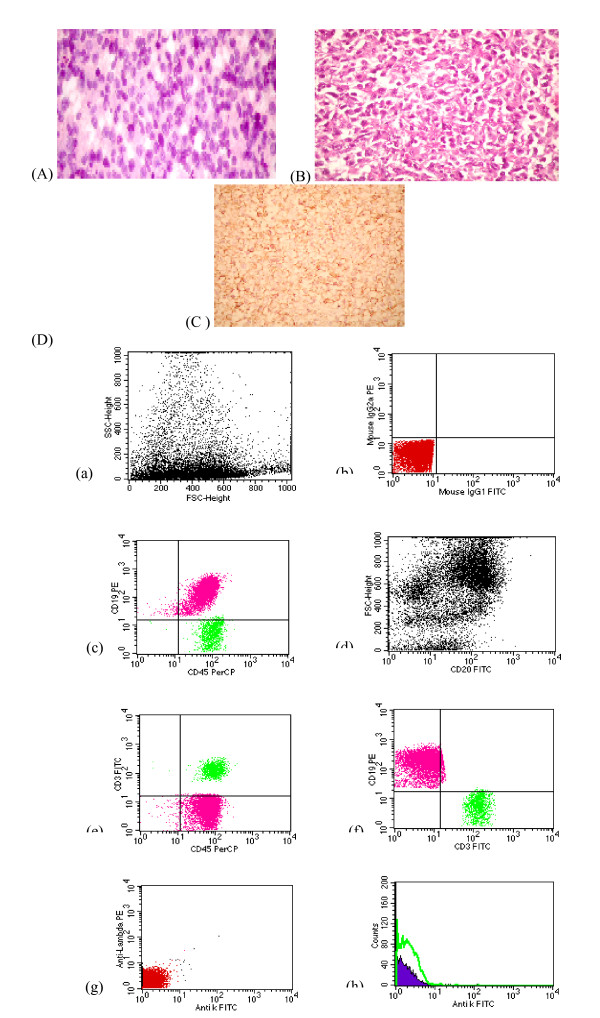
**(A) Touch imprint of DLBCL case showing large atypical lymphoid cells with vesicular nuclei and prominent nucleoli associated with atypical small to medium sized cells (modified PAP, 400×), (B): DLBCL case showing diffuse infiltration by large atypical lymphoid cells with vesicular nuclei and prominent nucleoli.** (H & E, 400×), (C): The same case showing positive reaction to CD 20 (400 ×), (D): FCM dot plots of a case of DLBCL showing (i) Large FSc, (ii) Negative isotype control (iii) Atypical large population showing positive reaction to CD45, CD 19, (iv) CD20 positive cells show large FSc (v&vi) Small population of CD 3 positive cells, (vii&viii) Absence of light chain expression by B-cells (LCR)

**Figure 2:**
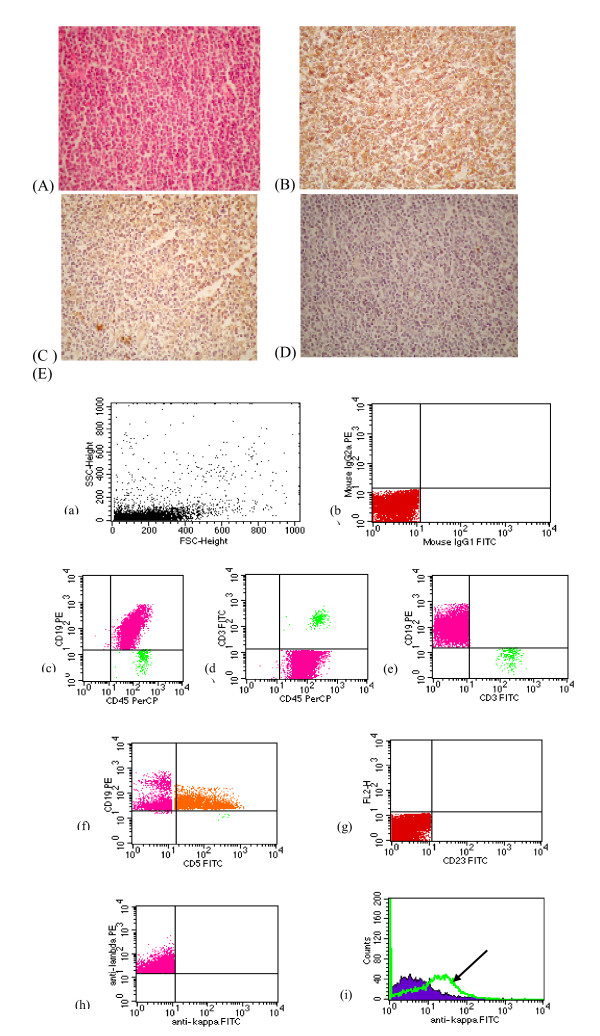
MCL case showing (A): Diffuse infiltration by monotonous atypical small lymphoid cells with dark nuclei and inconspicuous nucleoli (H & E, 400 ×), (B): Positive reaction to CD 20 (400×), (C): Positive reaction to CD 5(400×), (D): Negative reaction to CD23 (400×), (E): FCM dot plots of a case of MCL showing (i) Small FSc, (ii) Negative isotype control (iii, iv, v) Triple marker showing atypical population showing positive reaction to CD45, CD 19, negative reaction to CD 3 (vi) B-cells show positive reaction toCD 5, CD19 (vii) Negative reaction toCD 23 (viii) Monoclonality for λ light chain (ix) Overlapping histogram showing B-cell monoclonality expressing λ light chain (arrow).

Within the group of B cell NHL (32 cases), 24 cases (75%) were diffuse large B-cell lymphoma (DLBCL) showing positive reaction to CD45, CD20, CD19 and large FSc (Fig. 1), 2 cases (6.25%) were follicular lymphoma (FL) showing positive reaction to CD45, CD20, CD19, CD10, anti-BCL-2 and small to medium FSc. Three cases (9.4%) were small lymphocytic lymphoma (SLL) showing positive reaction to CD45, CD19, CD5, CD23 and small FSc, 2 cases (6.25%) were mantle cell lymphoma (MCL) showing positive reaction to CD45, CD19, CD5, negative reaction to CD23 and small FSc (Fig. 2), and a single case (3.12%) was diagnosed as T-cell rich B-cell lymphoma (TCRBCL) showing numerous T-cells which were CD45RO+ve, CD3+ve with small FSc. The less common B-cells in this case were CD20+ve and CD19+ve. The B-cells demonstrated large FSc and light-chain restriction.

**Figure 3:**
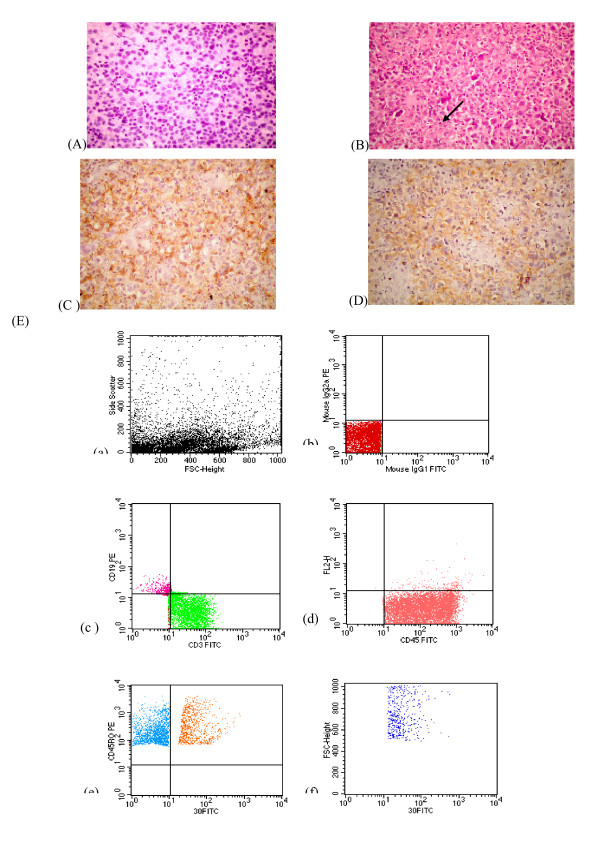
**(A):Touch imprint of ALTCL case showing large atypical lymphoid cells with vesicular nuclei and prominent nucleoli (modified PAP, 400 ×), (B): ALTCL case showing Diffuse infiltration by large atypical lymphoid cells with horse-shoe nuclei and prominent nucleoli.** Binucleated RS-like cells are encountered (arrow) (H & E, 400 ×)., (C) Malignant cells show positive reaction to CD 3, (D) Large anaplastic cells are positive for CD 30, (E): FCM dot plots of a case of ALTCL showing (i) Large FSc (ii) Negative isotype control (iii, iv) Atypical population showing positive reaction to CD45, CD 3, (v) Some of the CD45RO positive cells (T-cells)are positive for CD30 (vi) Positive cells for CD 30 have large FSc.

The 3 cases which were diagnosed as T-cell NHL were further subdivided into 2 cases (66.7%) of peripheral T-cell lymphoma (PTCL) showing positive reaction to CD45, CD3, CD45RO and a predominance of CD4+ve cells and a single case (33.3%) of anaplastic large T-cell lymphoma (ALTCL) with positive CD45, CD3, CD45RO and CD30 (Fig 3).

Light chain restriction (LCR) was detected in the 32 FCI-diagnosed B-cell lymphoma cases, where the ratio of κ/λ determined by FCM ranged from 0.14 to 15.67. Two out of these 32 cases (6.25%) showed absence of both κ & λ expression. A total of20 cases (62.5%) revealed monoclonality of κ-light chain with a mean of 5.73. The remaining 10 cases (31.25%) showed monoclonal expression of λ-light chain with a mean of 0.36. On the other hand, the κ/λ in the 9 RH cases ranged from 0.57 to 1.09 with a mean of 0.91 and they were all polyclonal.

### Histopathologic data

The frequency distribution of the 50 cases evaluated by histopathology/IHC was as follows: 4 cases (8%) were diagnosed as RH, 34 (68%) were B-cell NHL, 3 (6%) were T-cell NHL and 5 (10%) were HL. However, 4 cases (8%) were confirmed as metastatic duct carcinoma of breast origin (Table 1). Accordingly, NHL represented 88.1% of the diagnosed malignant lymphoma cases and HL represented 11.9% only with a ratio of 7.4: 1.

As regards to sub-typing by IHC of the 34 cases of B-cell NHL: 23 cases (67.65%) were DLBCL, 3 (8.82%) were FL, 2 (5.88%) were MCL, 3 (8.82%) were SLL and 3 cases (8.82%) were diagnosed as TCRBCL. On the other hand, the 3 cases diagnosed as T-cell NHL by IHC were subdivided into one case (33.3%) of PTCL and 2 cases (66.7%) were ALCL.

Light chain expression by B-cell NHL cases was not evaluated by immunohistochemistry, hence, it was not reported in patients' records.

### The correlations between FCI, histopathology and IHC

Seven groups were identified in the present study according to the data obtained from FCI, histopathology/IHC: **A) **4 cases were diagnosed as RH by FCI and were morphologically benign by histopathology; of which 3 were proved to be follicular hyperplasia and one case was diagnosed as sinus histiocytosis, **B) **2 cases were diagnosed as RH by FCI but they were categorized as NHL by histopathology/IHC (both cases proved to be TCRBCL), **C) **3 cases were diagnosed as RH by FCI and were proved to be HL by histopathology/IHC, **D) **4 cases showed NLT by FCI and were diagnosed as metastatic tumors by histopathology/IHC, **E) **32 cases were accurately diagnosed as B-cell NHL lymphoma by both FCI and histopathology/IHC, **F) **3 cases were clearly defined as T-cell NHL lymphoma by both FCI as well as histopathology/IHC, and **G) **2 cases were diagnosed as HL by both FCI and histopathology/IHC.

**Figure 4:**
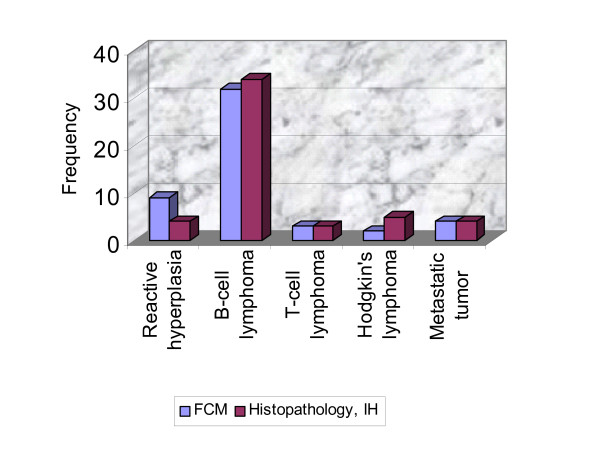
Frequency distribution histogram showing correlation between diagnostic groups in FCI and histopathology.

The overall concordance between FCI and histopathology/IHC was 88% (44/50 cases; Table 2 & Fig 4).

**Table 2:** Correlation between NHL sub-typing as characterized and diagnosed by FCI, routine histology and immunohistochemistry

**B-cell lymphoma subtypes**	**FCM diagnosed cases**	**Histopathology & Immunohistochemistry No. (%)**
**Diffuse large B-cell lymphoma**	24 (75)	23 (61.8)

***Follicular lymphoma***	2 (6.25)	3 (8.8)

***Small lymphocytic lymphoma***	3 (9.38)	2 (5.9)

***Mantle cell lymphoma***	2 (6.25)	3 (8.8)

***T-cell rich B-cell lymphoma***	1 (3.12)	3 (8.8)

***Total***	32(100)	34 (100)

**T-cell lymphoma subtypes**		

***Peripheral T-cell lymphoma***	2 (66.7)	1 (33.3)

***Anaplastic large T-cell lymphoma***	1 (33.3)	2 (66.7)

***Total***	3 (100)	3 (100)

### Correlation between the diagnosis of B-cell NHL and T-cell NHL subtypes by FCI and IHC

Within the group of B-cell NHL, it was possible to reach an accurate final diagnosis by FCI in 31/34 B-cell NHL cases representing 91.2% concordance. FCI showed 100% concordance with histopathology/IHC in diagnosing all cases of SLL and MCL (Fig 2). However, there was a difference in the other subtypes where FCI diagnosed 24 cases as DLBCL instead of 23 cases by histopathology/IHC. The extra case was proved to be FL by histopathology. Also, 2 cases were diagnosed as FL by FCI instead of 3 cases by IHC representing 66.7% concordance, and 1 case was diagnosed as TCRBCL by FCI instead of 3 cases by IHC representing 33.3% concordance. Within the group of T-cell NHL, there was 66.6% concordance between FCI and IHC (one case was diagnosed as PTCL and one case was diagnosed as ALTCL by both FCI and IHC, Fig. 3). However, one case was diagnosed as PTCL by FCI and was proved to be ALTCL by IHC.

### Statistical evaluation of FCI technique

Statistical evaluation of FCI technique included calculation of sensitivity, specificity, positive predictive value and negative predictive value. These calculations were performed to evaluate the application of FCI in diagnosis and characterization of NHL, HL, RH and NLT. Within the group of NHL the estimated sensitivity and specificity of FCI were 94.6% and 100%; respectively. The positive predictive value for diagnosis of NHL by FCI was 1 and the negative predictive value was 0.87. Within the group of HL, the sensitivity of FCI was 40% only (2 out of 5 HL cases were identified by FCI). However, the specificity was 100%. The positive predictive value was 1 and the negative predictive value was 0.94. Within the group of RH, the estimated sensitivity was 100% and the specificity was lower (89.1%). Positive predictive value was 0.44 and the negative predictive value was 1. As for the NLT, both sensitivity and specificity were 100%. Similarly, both positive predictive and negative predictive values were 1.

From the above results, FCI technique is 100% reliable in identifying NHL, HL and non-lymphomatous tumors as it gave no false positive results (specific) and to a lesser extent (89.1%) in diagnosing reactive hyperplasia. In addition, FCI technique is 100% reliable in excluding the diagnosis of reactive hyperplasia and non-lymphomatous tumors as it gave no false negative results (sensitive) and to a lesser extent (94.6%) in excluding NHL. However, FCI technique is not reliable in excluding the diagnosis of HL as its sensitivity is only 40%. This could be attributed to the small number of studied HL cases.

## Discussion

FCI has been applied in the diagnosis of lymphoma in lymph node biopsies as well as in extra-nodal sites. However, it requires fresh specimens to maintain viability and avoid loss of antigenicity through the process of fixation. So, processing of fresh tissue within minutes or few hours is mandatory [5].

In the present study we investigated the role of FCM in tissue diagnosis and classification of lymphoma. The results of the study showed 88% concordance between FCI aided by touch imprints and histopathology/IHC in the diagnosis of lymphoma. The diagnostic categories were NHL, HL, NLT and RH. Our results are consistent with Mandacova et al. [24] who found a concordance between FCI and histopathology in 89% of cases suspected to be lymphoma and Martinez et al. [25] who demonstrated that 218 out of 250 lymphoma cases (87.2%) were successfully diagnosed by FCI. A slightly higher concordance rate (94.1%) was reported by and Dunphy [5] who stated that FCI data were consistent with histopathologic diagnosis of 373 lymph node specimens examined.

A slightly lower percentage of concordance was estimated by Dong et al. [26] who assessed the ability of FCM to diagnose and sub-classify lymphoma in 139 cases and found that it was successful in 75% of the cases.

The role of FCI in diagnosing bone marrow infiltration by B-cell NHL was also assessed. Carulli et al. [27] reported that 89.5% of 114 cases studied were concordant in both FCI and histopathologic examination.

In the present work, we used a large panel of markers which resembles the panels utilized by other investigators [11,25,26,28]. It is also consistent with the panel established by the clinical cytometry society with the exception of the additional use of CD2 and CD7 for aberrant T-cell expression [10]. The large number of markers used in the present study was compromised by using dual and triple markers that detect simultaneous expression of 2 or 3 antigens on the same cell leading to a decrease in the number and quantity of used reagents and hence decrease in the cost/benefit value of the test.

In the present work, the studied cases were categorized into 7 groups (A-G) based on the results of FCI and histopathology/IHC. In the first group (A), FCI revealed 100% concordance with IHC in the sub-typing of all FCM-diagnosed B-cell lymphoma cases (32 cases) except for a single case which was diagnosed as DLBCL by FCI and was FL by histopathology although this case showed medium to large FSc denoting a large cell size By FCM and IHC was inconclusive for CD10 and negative for BCL-2.

In the second group, 3 cases were diagnosed as T-cell NHL by both FCI and IHC. These results were consistent with those of Chung et al. [29] who illustrated that T-cell surface markers are predominantly expressed in all T-cell lymphomas upon FCM analysis. However, Mayall et al. [28] mentioned that FCI is not helpful in the diagnosis of T-cell lymphoma since the CD4/CD8 ratio is not usually restricted in T cell lymphomas and loss of pan-T cell antigens was seen in some T-cell lymphomas. In their study, 4 out of 6 cases of T-cell lymphomas were successfully diagnosed by FCI whereas sub-typing was different in one case which was diagnosed as PTCL by FCI and ALTCL by IHC as it showed positive immunostaining for CD30. In FCI, this case was negative for CD30. However, this controversy in a single case can not be evaluated statistically due to small number of cases. Moreover, Jones et al. [30] demonstrated that CD30 is not expressed in 2% of ALTCL lymphoma cases.

In the third group 2 cases were successfully diagnosed as HL by both FCI and IHC whereas in the fourth group 3 cases were diagnosed as reactive hyperplasia by FCI and as HL, mixed cellularity type by histopathology. This could be attributed either to an error in the gating technique since the RS cells are CD45- and the chosen gate was adjusted to select CD45+ve cells only or due to loss of the large, fragile CD15+ cells during processing. Also touch imprint didn't show RS-like cells. The same results were reported by Witzig et al. [31] and Segal et al. [32] who also illustrated that the large neoplastic cells are fragile and are therefore lost during mechanical dispersion and preparation of the samples. In addition, **Braylan **[9] mentioned that the neoplastic cells in HL might escape detection by FCM due to their focal distribution in the lymph node.

In this regard, Young et al. [12] studied 107 FNA of suspected lymphoid lesions by cytology and FCM. Three out of these cases were HL but none of them showed RS cells in the FNA samples. On the other hand, FCI of these 3 cases showed polyclonal populations and they were diagnosed as RH. Similarly, Mayall et al. [28] and Dong et al. [26] provided evidence that FCI is not helpful in diagnosing HL since they identified HL by FCI in 33.3% only of cases and 44% (7 out of 16 HL cases) respectively. These data were confirmed by The Clinical Practice Guidelines for the Diagnosis and Management of Lymphoma which was approved by the National health and medical research council in Australia in 2005. According to these guidelines FCM findings are non-contributory in HL because the neoplastic cell population is scanty (1% of the total number of cells in suspension) and the neoplastic RS cells are CD45 negative.

In the fifth group, there was 100% concordance between FCI and histopathology/IHC results in non-lymphomatous tumors (4/4 cases). However, the exact typing of these tumors by FCM was not possible as it requires additional markers which were not used in this study. These results confirm the previously published data in this regard [12,15,16,28,33,19]. Similarly the sixth group showed a 100% concordance (4/4 cases) between FCI and histopathology/IH in the diagnosis of benign lymphoid lesions however the exact varieties of these lesions could not be categorized by FCI. Our results in this regard are in agreement with Jeffers et al. [34] who reported that 4 cases were diagnosed as RH by combining data from FNAC and FCI. Two cases were histopathologically diagnosed as RH and the other two cases were defined as Kikuchi's disease. Similarly, Laane et al. [35] demonstrated that 97% of 172 cases of reactive hyperplasia showed concordance between FCI was concordant with the morphologic diagnosis. However, Dunphy [16] mentioned that in hyperplastic disorders the classic morphologic criteria are considered more useful in the differential diagnosis.

In the current study 2 cases (4%) of pathologically-confirmed NHL were diagnosed as RH by FCM. Both cases were TCRBCL and the reason for the discordance was the presence of numerous mature T-cells and failure to detect the monoclonal B-cells population. This is consistant with the results of both Morse et al. [15] and Jeffers et al. [34].

Mayall et al. [28] showed that only 2 out of 73 cases were confusing because the cytology showed a malignant tumor with highly atypical cells mixed with benign looking lymphoid cells and the FCI showed no evidence of light chain restriction. However, paraffin embedded sections and immunohistochemistry showed that the large highly atypical cells were positive for CD45 and CD20 and negative for cytokeratin, S100, and CD3. Thus, a diagnosis of T-cell rich B-cell lymphoma was made. They assumed that the neoplastic B-cells were not strongly expressing light chains or they were masked by a population of reactive B-cells.

In this research, B-cell monoclonality was detected by FCI in 100% of FCM-diagnosed B-NHL however, these were not assessed by IHC. It has been reported that IHC does not usually provide a clear evidence of surface LCR due to the large amount of background immunoglobulin in the interstitial spaces of tissues which obscures the relatively weak monoclonal immunoglobulins on the surface of B-cells. The cell isolation and washing; usually required for FCM analysis; allows unambiguous assessment of light chain expression [19,34]. Similarly, Martinez et al. [25] showed that LCR was successfully detected in 90.5% of B-cell lymphoma cases (91 cases) whereas it was evaluated in 41.8% of the cases only by IHC.

In addition, Wu et al. [36] demonstrated that Ig light chain restriction was successfully detected by FCM in 15 of 17 88% of cutaneous B-cell lymphoma (primary or secondary), compared to 37% only by IHC. This was attributed to the fact nearly one-third of these cases were histologically suspicious but difficult lesions due to processing artifact, mixed cellular infiltrate, or paucity of abnormal cells. Moreover, Gomyo et al. [37] reported that FCI determined monoclonal B-cell population in 14 out of 92 (15.2%) bone marrow biobsies which were considered as negative samples by morphological examination.

In the present study, the estimated sensetivity and specificity of FCI in diagnosing NHL were 94.9% and 100%; respectively whereas the positive predictive value was 1 and the negative predictive value was 0.87. Comparable estimates were reported previously [26,25,38]. Das, [39] denoted that combined morphology & FCI has a sensitivity & specificity of 100%

Although recent studies demonstrated the significance of using modern techniques such as immunohistochemistry with tissue microarray [40], c-DNA microarray [41] and molecular pathology [42] in the proper diagnosis of lymphoma as well as in the prediction of patient's outcome. The use of FCM has the advantage of quick multiparametric analysis of a very large number of cells (20.000–50.000) cells and a better statistical representation of the population of interest. The choice of the appropriate antibody combinations, gating strategy and multiparametric analysis plays a key role in diagnosis of lymphoma [22].

FCM analysis offers another advantage over other techniques, where dual and tripple markers could be applied to detect co-expression of 2 or 3 antigens respectively on the same cell [43].

Also, FCM has the ability of precise detection of immunoglobulin light chain expression and so assessing the monoclonality of lymphoid populations [7,44].

FCI has been applied in the diagnosis of lymphoma in lymph node biopsies as well as in extranodal sites. However, it requires fresh specimens to maintain viability and avoid loss of antigenicity through tissue fixation, immediate suspension in chilled nutritional medium after surgical excision followed by processing of fresh tissue within minutes or few hours [5].

On the other hand, Immunohistochemistry on paraffin-embedded tissue is limited by poor antigen preservation and difficulties in defining antigens restricted to cell surfaces, being lost through fixation, which include the majority of lymphoid markers. Sufficient antibodies have been developed for the identification of B- and T-cell populations on paraffin-embedded tissue. Yet, immunoglobulin light chains are not reliably demonstrated on cell surfaces by this method [5].

It has been reported that IHC does not usually provide a clear evidence of surface light chain restriction due to the large amount of background immunoglobulin in the interstitial spaces of tissues which obscures the relatively weak monoclonal immunoglobulins on the surface of B-cells. The cell isolation and washing; usually required for FCM analysis; allows unambiguous assessment of light chain expression [19,35].

FCM is more sensitive than molecular techniques (RT-PCR, Q-RTPCR) as it avoids the probability of sample contamination by non neoplastic cells.

In addition, c-DNA array is rather costy and sophisticated tedious lengthy tests which can be performed in specialized labs only and needs high experience, special software system

We conclude that, the FCI contributes significantly to and are consistent with the final tissue diagnosis in the majority of our studied cases (88%). The false negative results of FCI could be attributed to the presence of heterogeneous populations of lymphocytes that might be present in special situations such as partial involvement of the lymphoid tissue by lymphoma cells, the presence of a follicular lymphoma with normal lymphoid cells in-between the neoplastic follicles, or the presence of numerous residual non-neoplastic lymphocytes among the neoplastic cells of diffuse lymphomas as in T-cell-rich B-cell lymphoma. Thus, in highly suspicious cases IHC is still required if no FCI abnormalities were detected. However, FCI has a definite role in detection of monoclonality (light chain) of NHL.

## Competing interests

The authors declare that they have no competing interests.     

## Authors' contributions

AM carried out the preparation of samples, acquisition of data, analysis and interpretation of data.   

AA carried out the correlation between FCI results and histopathologic/ IHC findings and drafted the manuscript.   

MH participated in the design of the study, supervision and revision of data interpretation and helped to draft the manuscript   

SMS conceived of the study, and participated in its design and revised the manuscript critically for important intellectual content.   

All authors read and approved the final manuscript.  
